# More older adults died at their preferred place after implementation of a transmural care pathway for older adults at the end of life: a before-after study

**DOI:** 10.1186/s12904-023-01218-0

**Published:** 2023-08-02

**Authors:** Iris van Doorne, Marike A. de Meij, Juliette L. Parlevliet, Vera M. W. van Schie, Dick L. Willems, Bianca M. Buurman, Marjon van Rijn

**Affiliations:** 1 Department of Internal Medicine, Section of Geriatric Medicine, Amsterdam University Medical Center, University of Amsterdam, Room D3-335, Meibergdreef 9, 1105AZ Amsterdam, The Netherlands; 2 Amsterdam Public Health, Aging and Later Life, Amsterdam, The Netherlands; 3 Center of Expertise Urban Vitality, Faculty of Health, Amsterdam University of Applied Science, Amsterdam, the Netherlands; 4 Palliative and Supportive Care Team, Oncology Center Amsterdam, OLVG, Oosterpark 9, Amsterdam, The Netherlands; 5 General Practice, Section of Medical Ethics, Amsterdam UMC Location University of Amsterdam, Meibergdreef 9, Amsterdam, The Netherlands; 6 Amsterdam UMC Location Vrije Universiteit, Medicine for Older People, Boelelaan 1117, Amsterdam, The Netherlands

**Keywords:** Palliative care, Transmural palliative care pathway, Before-after study

## Abstract

**Background:**

To improve transmural palliative care for older adults acutely admitted to hospital, the PalliSupport intervention, comprising an educational programme and transmural palliative care pathway, was developed. This care pathway involves timely identification of palliative care needs, advance care planning, multidisciplinary team meetings, warm handover, and follow-up home visits. With this study, we evaluate changes in patient-related outcomes and transmural collaboration after implementation of the care pathway.

**Methods:**

We conducted a before-after study, in which we compared 1) unplanned hospital admission and death at place of preference and 2) transmural collaboration before implementation, up to six months, and six to 18 months after implementation. Data from palliative care team consultations were collected between February 2017 and February 2020 in a teaching hospital in the Netherlands.

**Results:**

The palliative care team held 711 first-time consultations. The number of consultation, as well as the number of consultations for patients with non-malignant diseases, and consultations for advance care planning increased after implementation. The implementation of the pathway had no statistically significant effect on unplanned hospitalization but associated positively with death at place of preference more than six months after implementation (during/shortly after adjusted OR: 2.12; 95% CI: 0.84–5.35; p-value: 0.11, long term after adjusted OR: 3.14; 95% CI: 1.49–6.62; p-value: 0.003). Effects on transmural collaboration showed that there were more warm handovers during/shortly after implementation, but not on long term. Primary care professionals attended multidisciplinary team meetings more often during and shortly after implementation, but did not more than six months after implementation.

**Conclusions:**

The pathway did not affect unplanned hospital admissions, but more patients died at their place of preference after implementation. Implementation of the pathway increased attention to- and awareness for in-hospital palliative care, but did not improve transmural collaboration on long-term. For some patients, the hospital admissions might helped in facilitating death at place of preference.

**Supplementary Information:**

The online version contains supplementary material available at 10.1186/s12904-023-01218-0.

## Introduction

The early integration of palliative care has many benefits [1, 2]; it can increase quality of life, decrease symptom burden [3], and improve patients’ understanding of their prognosis, thereby improving their end-of-life decision making [4]. During hospital admissions, which are common for older patients at the end of life [5, 6], palliative care is primarily provided by non-specialists and a palliative care team can be consulted in case of complex situations [7]. Because these teams are usually not consulted until the last weeks of life [8], and communication and clarity about roles between healthcare professionals is often lacking [9] transmural palliative care for acutely hospitalized older adults is currently suboptimal. Transmural care was defined as a patient-centered care approach with close collaboration and joined responsibility between hospital and primary care organizations [10]. In the Netherlands, palliative care is provided according to the generalist plus specialist model: palliative care should be primarily provided by generalists who are not necessarily specialists in palliative care [7, 11]. In complex situations, palliative care specialists can be consulted, or can take over care if necessary. Since 2017, every Dutch hospital must have a palliative care team [12]. These palliative care teams can be consulted for advice on treating inpatients and outpatients at different stages of different diseases at the end of life.

To improve collaboration between hospitals and primary care organizations in the palliative care of acutely admitted older patients, the PalliSupport transmural palliative care pathway (hereafter referred to as pathway) was developed [13]. The pathway was developed based on research on the identification of palliative care needs and current collaboration [14–16], best practices, and in collaboration with experts in the field. The pathway involves timely identification of palliative care needs, advance care planning, multidisciplinary team meetings, warm handover, and follow-up home visits. Care is delivered by non-specialists in palliative care in collaboration with a transmural palliative care team, which includes both primary and secondary healthcare professionals. A pilot study was carried out including patients recruited during the initial 6 months in which the PalliSupport pathway was introduced (Table 1). In this study we found that adjustments were needed to incorporate a more active recruitment approach, additional training on identification and palliative care, further improvement on data collection and more involvement of general practitioners (GP) [13]. The PalliSupport pathway was continued as part of routine clinical care after the pilot study was completed.

**Table 1:** The PalliSupport pilot study

The palliative care team consisted of two clinical nurse specialists, a specialized general practitioner (GP), an oncologist, and GP trainees during their hospital internship. Patients were discussed during weekly team meetings. During the pilot study, the transitional care pathway was introduced. Researchers with a background in healthcare provided gave presentations on the early identification of patients with palliative care needs to nurses and physicians at the participating departments. Interactive training on how to initiate end-of-life conversations (in Dutch: STEM-training ^a^) was also offered to nurses and physicians. The goal was to increase knowledge on palliative care among non-specialists
The pilot study was a mixed-method feasibility study^b^. Patients were recruited between February 2018 and July 2018 from the department of pulmonology and gastroenterology in the OLVG teaching hospital in Amsterdam. Patients were screened for eligibility according to Supportive and Palliative Care Indicators Tool (SPICT) criteria. ^c^These criteria were unplanned hospital admission in the past six months, functional status, and malnutrition. The SPICT cut-off score for inclusion depended on the age of the patient. Patients aged 65–79 years with a score ≥ 2 and patients aged ≥ 80 years with a score ≥ 1 were eligible for inclusion. During the pilot study, eight patients received care according to the care pathway
Healthcare professionals were interviewed to evaluate the educational program and feasibility of the care pathway^c^ After the pilot study, the transitional palliative care team integrated parts of the care pathway into their daily practice. The care pathway was followed regardless of the hospital department/specialty from which the consultation was requested

^a^Oishi A, Murtagh FE. Palliat Med. 2014;28(9):1081–98

^b^van Wijmen MPS, Schweitzer BPM, Pasman HR, Onwuteaka-Philipsen BD. Fam Pract. 2020;37(5):641–7

^c^Flierman I, van Rijn M, de Meij M, et al. Pilot Feasibility Stud. 2020;6:129

To evaluate changes in patient-related outcomes and transmural collaboration after implementation of the care pathway, we performed this before-after study. Our aims were to determine changes in primary outcomes: 1) unplanned hospital admission and 2) death at place of preference, and secondary outcomes: transmural collaboration after implementation of the pathway. These insights could help to inform and improve future development and implementation of transmural palliative care interventions.

## Methods

We collected data from first-time palliative care team consultations held between February 2017 and February 2020 in an urban teaching hospital in Amsterdam with 633 beds. In this hospital, the PalliSupport intervention, comprising an educational programme and transmural palliative care pathway was implemented and the pilot study [13] took place (Table 1).

### Intervention

The pathway contained five key elements. These were:

- 1) Early identification of palliative care needs, using the SPICT criteria [17] and Surprise Question [18]
- 2) Systematic palliative assessment, based on the four domains of palliative care [19] and advance care planning after which an individual care plan was formulated. The assessment was conducted by a conversation between a member of the palliative care team and the patient and informal caregiver(s).
- 3) Team meetings, to which the patient’s own GP was invited. Individual care plans and the complexity assessments (based on symptom burden, patient needs, and whether the patient’s own GP could provide necessary care) were discussed. These assessments were based on the conversation the member of the palliative care team had with the patient.
- 4) The patient’s own GP received a warm handover, which was considered to be a conversation in person or by phone at hospital discharge.
- 5) A member of the transmural palliative care team visited the patient at home to follow up on the palliative assessment and adjust plans if needed (Table 2).
- During the pilot study patients were screened and recruited based on the SPICT criteria [17] and Surprise question [18]. After implementation all patients for whom the palliative care team was consulted could receive care according to the care pathway.

**Table 2:** An overview of the care pathway

Intervention	Components	Conducted by
Identification of palliative care needs during hospital admission	• Screening of palliative care needs based on SPICT criteria^a^ • Consulting the palliative care team	• Ward nurses and department physicians
Palliative care assessment and advance care planning	• Assessment of needs, preferences, and symptoms on physical, psychological, spiritual, and social level • Discussing treatment wishes, treatment limitations, and the patients’ preferred place of death^b^ • Formulating an individualized care plan^b^	• Department physician and/or palliative care team
Multidisciplinary team meeting	• Patients are discussed during weekly meetings of the transitional palliative care team, hospital specialists, and non-medical specialist • The patients’ own GP and community nurse are invited to the meeting (in person or by phone/videoconference)^a^ • The patients’ individual care plan is discussed^a^ • The complexity of the patients’ palliative care situation is assessed using a colour coding system indicating stability and severity of the situation^a^	• Department physician, patient’s own general practitioner, district nurse, palliative care team
Discharge	• The patient receives the individual care plan^a^ • Informal caregivers receive an information sheet about support^a^	• Department physician, ward nurse or palliative care team
Handover	• The patients’ GP is contacted prior to discharge or during multidisciplinary team meeting^b^ • A summary of the team meeting is sent to the patients’ GP and community nurse within 24 h after discharge^b^ • The medical handover is sent to the patients’ GP within 24 h after discharge^b^	• Department physician, ward nurse or palliative care team
Home visit and follow-up	• The patient is visited by a member of the transitional palliative care team^a^ • If needed, the patient is discussed during the team meeting, and the individualized care plan and colour code is adjusted^a^	• Palliative care team

^a^Elements that were completely new within the intervention

^b^Elements that were already performed for some patients but should be done for all patients during the study

A GP specialized in palliative care and district nurses were already part of the hospital-based palliative care team. This involvement increased during the implementation, meaning the primary care professionals of the palliative care team attended meetings more often and had more contact with other members. Individual care plans were formulated for patients and discussed with professionals who attended the multidisciplinary team meeting, to which the patient’s own GP and community nurses were invited. If the patient’s own GP could not attend to the meeting, a warm handover was conducted by phone. During team meetings, further involvement was determined according to a colour code (green, orange or red), which represented a ‘complexity assessment’ based on symptom burden, patient needs, and whether the patients’ own GP could provide the necessary care. Green indicated low complexity with no further input needed from the palliative care team; orange indicated disease progression and/or inadequate symptom management; and red indicated a highly complex and unstable situation. The patient, GP, and community nurse all received a paper or electronic copy of the care plan. The transmural palliative care team visited the patient at home after hospital discharge at least once, and more often if needed based on the colour code for complexity.

### Data collection

We collected data from all first-time palliative care team consultations conducted during the study period. A list of patients who received a consultation during this time period was provided and data were collected from electronic patient records. Data included patient characteristics, consultation characteristics, readmissions, and mortality.

### Outcomes

Primary outcomes were;

- Unplanned hospital admissions within six months after consultation (dichotomous). Unplanned hospital admissions was measured by collecting data from the electronic medical record in which hospital admissions are registered as elective or non-elective. Non-elective hospitalizations were registered as an unplanned hospitalization.
- Death at place of preference (dichotomous Yes/No), which was defined by the preferred place of death that was registered in the electronic medical record by a member of the palliative care team after their conversation with the patient. The actual place of death that was registered in the electronic medical record.

Secondary outcomes were changes in transmural collaboration: presence of the patient’s own primary healthcare professional (GP, district nurse, hospice resident) at team meetings, handover by phone, and follow-up home visits.

### Independent variables

Independent variables were age, gender, the main diagnosis (malignant or non-malignant), WHO/ECOG performance status, estimated life-expectancy, admission ward, preferred place of death, place of death, time until death after consultation, and reason for consultation.

### Statistical analysis

We analyzed the association between implementation of the pathway and the dependent variables. The implementation was divided in three phases: 1) pre-implementation phase: one year period before implementation started (Feb 2017 – Jan 2018), 2) during/shortly after implementation phase: six months period of the implementation and six months after implementation (Feb 2018 – Jan 2019), and 3) long term after implementation phase: one year period that started six months after the implementation ended (Feb 2019 – Jan 2020). The pre-implementation phase was used as a reference category. We analyzed changes on the individual patient level, and in every implementation phase, data from new patients were collected. Consequently, patients in the different implementation phases were not the same and data were not dependent. We performed multivariable logistic regression analysis to calculate and report adjusted odds ratios (OR) with corresponding 95% confidence intervals (CI). For each outcome, the included patients were tested for differences between implementation groups. Variables with statistically significant differences were discussed to assess whether these were relevant co-variables and if so, included in multivariable logistic regression analysis.

A *p*-value < 0.05 was considered statistically significant. All statistical analyses were done using IBM SPSS Statistics Version 26.

We used the STROBE guidelines [20] to report our results.

## Results

We analysed 711 consultations conducted between February 2017 and February 2020 (212 pre-implementation, 248 during/shortly after implementation, 251 long term after implementation). For patients for whom the palliative care team was consulted more than once, we included patients based on the first consult and considered other consultations for this patient as follow-up.

### Patients and consultations

Patients had a mean age of 71.6 years (SD 12.6) and 50.4% were male. Most patients were diagnosed with cancer (71.3%), however, the proportion of consultations for patients with non-malignant diseases increased after implementation (pre-implementation: 20.8%, during/short-term: 32.7%, long-term: 31.5%). The majority of patients had a WHO/ECOG performance status of 3 (39.9%) or 4 (26.3%) and the estimated life expectancy was less than three months (30.5%). Most consultations were for patients admitted to the department of pulmonology or cardiology (36.9%) Almost half of all consultations (43.4%) were requested for advance care planning and/or guidance in the upcoming future. After implementation, the number of consultations for advance care planning did not change, but the number of consultations for advice in the dying phase decreased. The preferred place of death was discussed less often during/shortly after implementation, but more often long term after implementation (pre-implementation: 54.2%, during/shortly after implementation: 24.2%, long term after implementation: 58.2%) (Table 3).

**Table 3:** Baseline characteristics

	Total *N* = 711	Pre-implementation *N* = 212	During/short-term after implementation *N* = 248	Long-term after implementation *N* = 251	*P*-value
Male, N (%)	358 (50.4)	109 (51.4)	125 (50.4)	124 (49.4)	0.89^a^
Age, mean (SD)	71.6 (12.6)	70.5 (13.4)	72.0 (12.3)	72.0 (12.1)	0.67^b^
Diagnosis, N (%)					
Non-malignant diseases	204 (28.7)	44 (20.8)	81 (32.7)	79 (31.5)	**0.008**^a^
WHO/ECOG performance status, N (%)	*N* = 654	*N* = 199	*N *= 216	*N* = 240	**0.05**^a^
2: Ambulatory and capable of self-care but unable to carry out any work	140 (21.4)	43 (21.6)	47 (21.8)	50 (21.0)	
3: Capable of only limited self-care: confined to bed/chair more than 50% of waking hours	261 (39.9)	79 (39.7)	76 (35.2)	106 (44.2)	
4: Completely disabled	172 (26.3)	44 (22.1)	62 (28.7)	66 (27.5)	
Prognosis, N (%)	*N* = 693	*N* = 210	*N* = 236	*N* = 248	0.09^a^
Days to weeks	181 (26.1)	47 (22.4)	66 (28)	68 (27.4)	
< 3 months	212 (30.5)	65 (31.0)	62 (26.3)	85 (34.3)	
< 6 months and < 1 year	158 (22.8)	50 (23.8)	64 (27.1)	44 (17.7)	
> 1 year	20 (2.9)	7 (3.3))	6 (2.5)	7 (2.8)	
Difficult to make an estimation	123 (17.7)	41 (19.5)	38 (16.1)	44 (17.7)	
Admission department, N (%)	*N* = 583	*N* = 156	*N* = 212	*N* = 215	** < 0.001**^a^
Pulmonology/cardiology	215 (36.9)	47 (30.1)	78 (37.0)	90 (41.7)	
Internal medicine (both malignant and non-malignant internal diseases)	182 (31.2)	68 (43.6)	32 (15.2)	82 (38.0)	
Other^d^	186 (31.9)	41 (26.3)	101 (47.9)	43 (20.0)	
Reason for consultation	*N* = 684	*N* = 201	*N* = 234	*N* = 245	** < 0.001**^a^
Advance care planning and/or guidance in the upcoming process	331 (43.4)	103 (51.2)	100 (42.6)	128 (51.6)	
Advice on symptoms, medication	129 (18.9)	35 (17.4)	34 (14.5)	60 (24.2)	
Guidance in after care and support system	107 (15.6)	36 (17.9)	30 (12.8)	41 (16.5)	
Guidance/advice in the dying phase	117 (17.1)	27 (13.4)	71 (30.2)	19 (7.7)	
Preferred place of death discussed, N (%)	321 (45.1)	115 (54.2)	60 (24.2)	146 (58.2)	**0.02**^a^
Home	160 (49.8)	64 (55.7)	34 (56.7)	62 (42.5)	
Hospital	21 (6.5)	6 (5.2)	8 (13.3)	7 (4.8)	
Care facility (care home / hospice)	94 (29.3)	29 (25.2)	17 (28.3)	48 (32.9)	
No clear place mentioned	46 (14.3)	16 (13.9)	1 (1.7)	29 (19.9)	
Time until death after consultation (days), Median [IQR] *N* = 557	18.46 [4.62 – 65.77]	26.5 [8.1 – 92.0]	12.3 [3.5 – 47.3]	18.5 [4.6 – 61.2]	**0.004**^c^
Place of death, N (%)	*N* = 484	*N* = 118	*N* = 161	*N* = 202	0.06^a^
Home	135 (27.9)	30 (25.4)	44 (27.3)	60 (29.7)	
Hospital	250 (51.7)	65 (55.1)	93 (57.8)	90 (44.6)	
Care facility (care home / hospice)	99 (20.5)	7 (5.9)	4 (2.5)	8 (4.0)	
Death at place of preference	*N* = 208 121 (58.2)	*N* = 61 25 (41,0)	*N* = 44 26 (59,1)	*N* = 103 70 (68.0)	**0.003**^a^
Hospital (re)admission within six months after consultation	*N* = 522 121 (23.2)	*N* = 171 41 (24.0)	*N* = 175 43 (24.6)	*N* = 175 37 (21.1)	0.46^a^
Consult with multidisciplinair team meeting, N (%)	547 (76.9)	163 (76.5)	181 (73.0)	203 (81.5)	0.12^a^
At least one of the patients’ own primary care professional attended the multidisciplinair team meeting, N (%)	271 (49.5)	80 (49.1)	129 (71.3)	62 (30.5)	** < 0.001**^a^
Patient’s own GP attended the multidisciplinary team meeting, N (%)	151 (23.7)	63 (38.7)	56 (30.9)	32 (15.8)	** < 0.001**^a^
Warm handover to GP (by member of palliative care team or ward physician), N (%)	259 (36.5)	81 (34.6)	95 (38.9)	95 (34.7)	**0.002**^a^
Home visit, N (%)	15 (2.9)	1 (0.6)	7 (4.0)	7 (4.0)	0.14^a^

^a^Chi-squared test

^b^One-way ANOVA

^c^Kruskall-Wallis test

^d^other admission wards were: gynaecology, nephrology, urology, surgery, intensive care unit, orthopaedics, geriatrics

### Key elements of the care pathway

There were 638 patients included to analyze changes in transmural collaboration. Attendance of the patient’s own primary care professionals (GP and/or district nurse) at team meetings was increased during/shortly after implementation, but decreased long term after implementation (pre-implementation: 49.1%, during/shortly after implementation: 71.3%, long term after implementation: 30.5%). Attendance of the patients’ own GP at team meetings decreased after implementation (pre-implementation 38.7%, during/shortly after implementation: 30.9%, long term after implementation: 15.8%). Warm handovers were done a little more often after implementation (pre-implementation: 34.6%, during/shortly after implementation: 38.9%, long term after implementation: 34.7%). Pre-implementation, one follow-up home visit was done, during/shortly after implementation as well as long term after implementation seven visits to the patients home were made (Table 3).

### Unplanned hospital admission after consultation

Patient who did not die during the hospital admission at which the palliative care team was first consulted were included in the logistic regression analysis for unplanned hospital admission, leaving 522 patients included in this analysis, of which 23.2% experienced an unplanned hospital admission within six months after consultation (Additional file 1: Appendix 1). We found statistically significant differences between implementation groups for the factors: malignant/non-malignant diseases, admission department, reason for consultation and actual place of death. Because the actual place of death was not considered as a confounder, but as a consequence of a hospital admission, we included the reason for consultation, malignant/non-malignant diseases, and admission department in the multivariable logistic regression analysis. Adjusted logistic regression analysis showed no statistically significant effects of the implementation (during/shortly after implementation adjusted OR: 1.23; 95% CI: 0.67–2.26; p-value 0.51; long-term after implementation adjusted OR: 0.79; 95% CI: 0.44–1.41; *p*-value: 0.42) (Table 4).

**Table 4:** Univariable and multivariable logistic regression analysis of the association between implementation of the PalliSupport intervention and unplanned (re)admission

	Univariable logistic regression analysis			Multivariable logistic regression analysis		
	OR	95% CI	*P*-value	OR	95% CI	*P*-value
During/shortly after implementation^a^	1.09	0.67–1.79	0.73	1.23	0.67–2.26	0.51
Long term after implementation^a^	0.86	0.52–1.43	0.56	0.79	0.44 – 1.41	0.42
Malignant disease				1.63	0.94 – 2.83	0.08
Admission department: Internal medicine^b^				**2.26**	**1.27 – 4.02**	**0.006**
Admission department: Other^b^				0.85	0.43 – 1.69	0.65
Reason for consultation: Advice on symptom management/medication^c^				0.87	0.47 – 1.61	0.65
Reason for consultation: guidance in after care and support system^c^				0.75	0.42 – 1.35	0.34
Reason for consultation: Guidance/advice in the dying phase^c^				**0.12**	**0.03 – 0.38**	** < 0.001**

^a^Reference category: Before implementation

^b^Reference category: Pulmonology/cardiology

^c^reference category: Advance care planning/guidance in the upcoming process

### Mortality and death at place of preference

Most patients died within one year after consultation (88%) with a median of 18.5 days. We collected data on the preferred and actual place of death for 208 patients (Additional file 2: Appendix 2). Of these patients, 58.2% died at their preferred place. Within the sample of 208 patients, we found statistically significant differences between implementation groups for admission department, reason for consultation, preferred place of death. All three variables were included in multivariable logistic regression analysis. During/shortly after implementation, we found no statistically significant change in death at place of preference (adjusted OR: 2.12; 95% CI: 0.84–5.35; p-value: 0.11). Long term after implementation, the pathway was positively associated with death at place of preference (adjusted OR: 3.14; 95% CI: 1.49–6.62; p-value: 0.003) (Table 5).

**Table 5:** Univariable and multivariable logistic regression analysis of the association between implementation of the PalliSupport intervention and death at place of preference

	Univariable logistic regression analysis			Multivariable logistic regression analysis		
	OR	95% CI	*P*-value	OR	95% CI	*P*-value
During/shortly after implementation^a^	2.22	2.12	0.84 – 5.35	2.12	0.84 – 5.35	0.11
Long term after implementation^a^	2.17	**3.14**	**1.49 – 6.62**	**3.14**	**1.49 – 6.62**	**0.003**
Admission department: Internal medicine^b^				0.85	0.46 – 1.91	0.85
Admission department: Other^b^				0.88	0.39 – 1.99	0.76
Reason for consultation: Advice on symptom management/medication^c^				1.33	0.52 – 3.40	0.56
Reason for consultation: guidance in after care and support system^d^				1.63	0.74 – 3.57	0.23
Reason for consultation: Guidance/advice in the dying phase^d^				0.79	0.27 – 2.32	0.67
Preferred place of death: hospital^d^				**3.38**	**1.09 – 10.54**	**0.04**
Preferred place of death: care facility^d^				2.80	0.29 – 26.96	0.37

^a^Reference category: Before implementation

^b^Reference category: Cardiology/pulmonology

^c^Reference category: Advance care planning/guidance in the upcoming process

^d^Reference category: Home

## Discussion

We analyzed all first-time palliative care consultations conducted before, during, and after implementation of the PalliSupport pathway and evaluated the changes after implementation on patient-related outcomes and transmural collaboration. We observed changes in transmural collaboration during/shortly after implementation, but not so much long term after implementation. Therefore, we believe the observed changes in the primary outcome preferred place of death in the long term are not caused by changes in transmural collaboration, but by a combination of factors, which were the education for non-specialists, increased attention to palliative care, more focus on transmural collaboration, and time. The palliative care team in this study already involved primary care professionals in team meetings. Therefore, It is possible that implementation will lead to stronger changes in other teams with less focus on transmural collaboration.

### Interpretation of findings

Our study only showed improvement in transmural collaboration during/shortly after implementation, indicating the team had a more hospital-based approach. At least one of the patients’ own primary healthcare professionals attended more meetings during/shortly after implementation, but not on the long term. This could be caused by practical issues such as travel time [13], and the COVID-19 pandemic which could have made it less likely to visit other healthcare organizations. Moreover, the division between primary and secondary care, and perceptions on were palliative care should be provided [21] probably influenced the involvement of primary care professionals, especially GPs.

While a systematic review of Saunders et al. [6] suggested that palliative care involvement during hospital admission might decrease readmission rates, we found no association between implementation and (re)admission, in line with results of another transmural care pathway [22]. Members of the palliative care team felt responsible for the patients under discussion, and patients and GPs expected the palliative care team to become the main contact [13]. Consequently, patients and GPs might have had the tendency to reach out the hospital sooner in case of problems at home, leading to more hospital admissions. The pathway aimed to avoid hospital admission with the following key elements: [23] recognizing the approach of death, discussions about treatment wishes and symptoms, and monitoring through follow-up home visits. However, the transmural palliative care team still got involved mostly in the last weeks to months of life, limiting the time to discuss and prepare for the approach of death and to fix problems such as limited support systems, small houses with high stairs, and limited home care [24, 25]. These problems cannot be fixed by individual hospitals or professionals and require large scale improvement strategies on the social or (community) political level. In addition, the number of performed home-visits was low, which could indicate insufficient monitoring after discharge. The number of consultations before and after implementation requested for advance care planning remained equal, however, it could be possible that non-specialist performed more advance care planning discussions themselves after the educational program. This had no effect on the prevention of hospital admissions, but might have contributed to the effect on death at place of preference.

Overall, half of the patients in our study died at their place of preference, which is in line with previous research [26]. Our results showed that long term after implementation was associated positively with death at place of preference. A systematic review comparing different transmural palliative care approaches revealed that a hospital-based approach was most effective in facilitating death at home [27], which could explain why we found an effect on death at place of preference. We hypothesize that patient-level effects are not the result of individual elements of the pathway, but of education, increased attention for palliative care and the pathway as a whole including a change of culture in the hospital. This is supported by the increased number of consultations for specialist palliative care. There were also more requests for patients with non-malignant diseases. This demonstrates the importance of gaining knowledge about recognition of palliative care needs, which is considered to be difficult especially for patients with non-malignant diseases [14]. Also, long term after implementation the preferred place of death was discussed more often, which might indicate more attention for patients preferences. Since unplanned hospital admissions were not reduced, we think that for some patients, these admissions eventually made death at the place of preference possible because of better symptom management and time for arranging care at home, in care homes, and in hospices [28]. We found that on long term after implementation, especially the number of patient preferring and actually dying in hospices increased. This could be caused by an overall increase in hospice care [29], but could also be caused by the involvement of the palliative care team, [30] which might have led to more awareness of hospice care among patients.

### Strengths and limitations

A strength of this study is that we collected data from all new consultations conducted with the palliative care team before, during, and after implementation of the pathway, which provides insight into the short-term and long-term effects of implementation.

This study also has limitations, and therefore the interpretation of the changes we found should be made with caution. A limitation of this study is the retrospective nature of the study design. Because of that, changes in patient-related outcomes could have also been caused by other factors, which we did not measure. The patients that were recruited during the pilot study were screened for SPICT criteria and the surprise question while other patients were not. This could have caused differences between these groups, however, only eight patients were recruited during the pilot study [13]. We do acknowledge that a randomized controlled trial is the gold standard approach to evaluating effectiveness [31]. However, given the complex and time-consuming nature of improving transmural palliative care [32], we felt a before-after design would be the best alternative [33]. All data were collected from electronic patient records, which were based on reports made by the patients’ caregivers and members of the palliative care team. Consequently, we could not collect data about conversations and contacts that were not reported in the electronic patient record, which might have caused an underestimation of the transmural collaboration. Also, we could not collect data of hospital admissions in other hospitals. Considering the sample size, we could not include all possible relevant factors in the multivariable logistic regression analysis, but only included statistically significant factors. Future studies should involve prospective longitudinal data collection and collect data on symptom management, patient satisfaction with care, the number of care transitions and other patient-related outcomes.

In conclusion, our study shows that implementation of the pathway did not result in improvement of all patient-related outcomes and transmural collaboration. The pathway did not affect unplanned hospital admissions, but more patients died at their preferred place after implementation. The attention and awareness for in-hospital palliative care increased, but did not improve transmural collaboration on long term. For some patients, the hospital admissions might have helped in facilitating death at place of preference.

## Supplementary Information

**Additional file 1:Appendix 1.** Baseline characteristics study sample included in analysis for hospital (re)admission.

**Additional file 2:Appendix 2.** Baseline characteristics study sample included in analysis for death at place of preference.

## Data Availability

The datasets used and analysed during the current study are available from the corresponding author on reasonable request.
