# Supplementary material for: More older adults died at their preferred place after implementation of a transmural care pathway for older adults at the end of life: a before-after study
Source: BMC Palliat Care. 2023 Aug 2;22:110. doi: 10.1186/s12904-023-01218-0 (PMC10394846; doi:10.1186/s12904-023-01218-0)
Supplement: Supplementary file 1 — Additional file 1:Appendix 1. Baseline characteristics study sample included in analysis for hospital (re)admission. [file 12904_2023_1218_MOESM1_ESM.docx]

Appendix 1. Baseline characteristics study sample included in analysis for hospital (re)admission

|  | Total  N=522 | Pre-implementation  N=171 | During/short-term after implementation  N=175 | Long-term after implementation  N=176 | P-value |
| --- | --- | --- | --- | --- | --- |
| Male, N (%) | 261 (50.0) | 88 (51.5) | 88 (50.3) | 85 (48.3) | 0.83 ^a^ |
| Age, mean (SD) | 70.0 (12.3) | 70.6 (13.1) | 70.1 (12.4) | 69.3 (11.5) | 0.62 ^b^ |
| Diagnosis, N (%) |  |  |  |  | **0.02** ^a^ |
| Non-malignant diseases | 119 (22.8) | 26 (15.2) | 46 (26.3) | 47 (26.7) |  |
| WHO/ECOG performance status, N (%) |  |  |  |  |  |
| 2: Ambulatory and capable of self-care but unable to carry out any work | 127 (26.1) | 38 (23.9) | 43 (27.0) | 46 (27.4) |  |
| 3: Capable of only limited self-care: confined to bed/chair more than 50% of waking hours | 211 (43.4) | 71 (44.7) | 58 (36.5) | 82 (48.8) |  |
| 4: Completely disabled | 68 (14.0) | 17 (10.7) | 27 (17.0) | 24 (14.3) |  |
| Prognosis, N (%) |  |  |  |  | 0.08 ^a^ |
| Days to  weeks | 70 (13.6) | 23 (13.6) | 29 (17.0) | 18 (10.4) |  |
| < 3 months | 172 (33.5) | 54 (32.0) | 49 (28.8) | 69 (39.7) |  |
| < 6 months and  < 1 year | 146 (28.4) | 49 (39.0) | 56 (33.0) | 41 (23.5) |  |
| > 1 year | 20 (3.9) | 7 (4.1) | 6 (3.5) | 7 (4.0) |  |
| Difficult to make an estimation | 105 (20.5) | 36 (21.3) | 30 (17.6) | 39 (22.4) |  |
| Admission department, N (%) | N=397 | N=114 | N=141 | N=142 | **<0.001** ^a^ |
| Pulmonology/cardiology | 152 (38.3) | 37 (32.5) | 53 (37.6) | 62 (43.7) |  |
| Internal medicine (both malignant and non-malignant internal diseases) | 126 (31.7) | 47 (41.2) | 20 (14.2) | 59 (41.5) |  |
| Other ^d^ | 119 (30.0) | 30 (26.3) | 68 (48.2) | 21 (14.8) |  |
| Reason for consultation |  |  |  |  | **<0.001** ^a^ |
| Advance care planning and/or guidance in the upcoming process | 266 (51.7) | 89 (52.7) | 75 (44.1) | 102 (58.0) |  |
| Advice on symptoms, medication | 91 (17.7) | 24 (14.2) | 30 (17.6) | 37 (21.0) |  |
| Guidance in after care and support system | 107 (20.8) | 46 (27.2) | 25 (23.5) | 36 (20.5) |  |
| Guidance/advice in the dying phase | 51 (9.9) | 10 (5.9) | 40 (23.5) | 1 (0.6) |  |
| Preferred place of death discussed, N (%) | N=277 | N=86 | N= 41 | N=100 | 0.31 ^a^ |
| Home | 143 (63.0) | 59 (68.6) | 28 (68.3) | 56 (56.0) |  |
| Hospital | 9 (4.0) | 5 (5.8) | 1 (2.4) | 3 (3.0) |  |
| Care facility (care home / hospice) | 75 (33.1) | 22 (25.6) | 12 (29.2) | 41 (41.0) |  |
| Time until death after consultation (days), Median [IQR] | 37 [15-85.8] | 49 [15 – 104] | 30 [14 – 80] | 35.5 [16 – 74] | 0.37 ^c^ |
| Place of death, N (%) | N=293 | N=76 | N=88 | N=129 | **0.04** ^a^ |
| Home | 134 (45.7) | 30 (39.5) | 44 (50.0) | 60 (46.5) |  |
| Hospital | 60 (20.5) | 23 (30.3) | 20 (22.7) | 17 (13.2) |  |
| Care facility (care home / hospice) | 99 (33.8) | 23 (30.3) | 24 (27.2) | 52 (40.3) |  |

^a^ Chi-squared test

^b^ One-way ANOVA

^c^ Kruskall-Wallis test

^d^ other admission wards were: gynaecology, nephrology, urology, surgery, intensive care unit, orthopaedics, geriatrics
