# Supplementary material for: More older adults died at their preferred place after implementation of a transmural care pathway for older adults at the end of life: a before-after study
Source: BMC Palliat Care. 2023 Aug 2;22:110. doi: 10.1186/s12904-023-01218-0 (PMC10394846; doi:10.1186/s12904-023-01218-0)
Supplement: Supplementary file 2 — Additional file 2:Appendix 2. Baseline characteristics study sample included in analysis for death at place of preference. [file 12904_2023_1218_MOESM2_ESM.docx]

Appendix 2. Baseline characteristics study sample included in analysis for death at place of preference

|  | Total  N=208 | Pre-implementation  N=61 | During/short-term after implementation  N=44 | Long-term after implementation  N=103 | P-value |
| --- | --- | --- | --- | --- | --- |
| Male, N (%) | 109 (52.4) | 35 (57.4) | 18 (40.9) | 56 (54.4) | 0.21 ^a^ |
| Age, mean (SD) | 71.0 (11.9) | 72.7 (11.6) | 71.1 (12.9) | 69.7 (11.7) | 0.34 ^b^ |
| Diagnosis, N (%) |  |  |  |  | 0.67 ^a^ |
| Non-malignant diseases | 45 (21.6) | 11 (18.0) | 11 (25.0) | 23 (22.3) |  |
| WHO/ECOG performance status, N (%) |  |  |  |  | 0.19 ^a^ |
| 2: Ambulatory and capable of self-care but unable to carry out any work | 35 (17.3) | 10 (16.9) | 7 (14.3) | 18 (17.8) |  |
| 3: Capable of only limited self-care: confined to bed/chair more than 50% of waking hours | 99 (49.0) | 30 (50.8) | 17 (40.5) | 52 (51.5) |  |
| 4: Completely disabled | 56 (27.7) | 17 (28.8) | 12 (28.6) | 27 (26.7) |  |
| Prognosis, N (%) |  |  |  |  | 0.38 ^a^ |
| Days to  weeks | 63 (30.3) | 24 (39.3) | 13 (29.6) | 26 (25.2) |  |
| < 3 months | 96 (46.2) | 22 (36.1) | 21 (47.7) | 53 (51.5) |  |
| < 6 months and  < 1 year | 33 (15.9) | 10 (16.4) | 7 (15.9) | 16 (15.5) |  |
| > 1 year | 1 (0.5) | 1 (1.6) | - | - |  |
| Difficult to make an estimation | 15 (7.2) | 4 (6.6) | 3 (6.8) | 8 (7.8) |  |
| Admission department, N (%) | N= 188 | N=56 | N=39 | N=93 | **0.02** ^a^ |
| Pulmonology/cardiology | 60 (31.9) | 15 (26.8) | 17 (43.6) | 28 (30.1) |  |
| Internal medicine (both malignant and non-malignant internal diseases) | 88 (46.8) | 29 (51.8) | 9 (23.1) | 50 (53.8) |  |
| Other ^d^ | 40 (21.3) | 12 (21.4) | 13 (33.3) | 15 (16.1) |  |
| Reason for consultation |  |  |  |  | **< 0.001** ^a^ |
| Advance care planning and/or guidance in the upcoming process | 106 (51.5) | 23 (38.3) | 18 (41.9) | 65 (63.1) |  |
| Advice on symptoms, medication | 27 (13.1) | 9 (15.0) | 3 (7.0) | 15 (14.6) |  |
| Guidance in after care and support system | 49 (23.8) | 21 (35.0) | 7 (16.3) | 21 (20.4) |  |
| Guidance/advice in the dying phase | 24 (11.7) | 7 (11.7) | 15 (34.9) | 2 (1.9) |  |
| Preferred place of death discussed, N (%) |  |  |  |  | 0.45 ^a^ |
| Home | 115 (55.3) | 38 (62.3) | 22 (50.0) | 54 (52.4) |  |
| Hospital | 22 (10.6) | 6 (9.8) | 8 (18.2) | 8 (7.8) |  |
| Care facility (care home / hospice) | 71 (34.2) | 17 (27.9) | 14 (31.8) | 40 (38.8) |  |
| Time until death after consultation (days), Median [IQR] | 14.5 [6 – 42] | 12.5 [6 – 63] | 14 [6 – 31.8] | 17.5 [6.3-43] | 0.63 ^c^ |
| Place of death, N (%) |  |  |  |  | **0.02** ^a^ |
| Home | 75 (36.1) | 18 (29.5) | 15 (34.1) | 42 (40.8) |  |
| Hospital | 77 (37.0) | 29 (47.5) | 22 (50.0) | 26 (25.2) |  |
| Care facility (care home / hospice) | 56 (27.0) | 14 (22.9) | 7 (15.9) | 35 (34.0) |  |

^a^ Chi-squared test

^b^ One0way ANOVA

^c^ Kruskall-Wallis test

^d^ other admission wards were: gynaecology, nephrology, urology, surgery, intensive care unit, orthopaedics, geriatrics
